# Supplementary material for: Familial Hypercholesterolemia in Premature Acute Coronary Syndrome. Insights from CholeSTEMI Registry
Source: J Clin Med. 2020 Oct 29;9(11):3489. doi: 10.3390/jcm9113489 (PMC7692119; doi:10.3390/jcm9113489)
Supplement: Supplementary file 1 [file jcm-09-03489-s001.pdf]

**Table S1: Regions covered by the NGS panel.**

| Gene | Chr | Chr Start | Chr End | # Amplicons | Total Bases | Covered Bases | Missed Bases | Coverage (%) |
|---|---|---|---|---|---|---|---|---|
| APOB | chr2 | 21251194 | 21251415 | 2 | 222 | 222 | 0 | 100 |
| APOB | chr2 | 21224597 | 21226211 | 10 | 1615 | 1615 | 0 | 100 |
| APOB | chr2 | 21227947 | 21235528 | 43 | 7582 | 7582 | 0 | 100 |
| APOB | chr2 | 21250695 | 21250942 | 2 | 248 | 248 | 0 | 100 |
| APOB | chr2 | 21252765 | 21252892 | 1 | 128 | 128 | 0 | 100 |
| APOB | chr2 | 21256166 | 21256395 | 2 | 230 | 230 | 0 | 100 |
| APOB | chr2 | 21257683 | 21257778 | 1 | 96 | 96 | 0 | 100 |
| APOB | chr2 | 21227136 | 21227329 | 1 | 194 | 194 | 0 | 100 |
| APOB | chr2 | 21237940 | 21238137 | 1 | 198 | 198 | 0 | 100 |
| APOB | chr2 | 21249655 | 21249841 | 1 | 187 | 187 | 0 | 100 |
| APOB | chr2 | 21258451 | 21258585 | 1 | 135 | 135 | 0 | 100 |
| APOB | chr2 | 21259967 | 21260132 | 1 | 166 | 166 | 0 | 100 |
| APOB | chr2 | 21266731 | 21266822 | 1 | 92 | 92 | 0 | 100 |
| APOB | chr2 | 21242590 | 21242782 | 1 | 193 | 193 | 0 | 100 |
| APOB | chr2 | 21247800 | 21248001 | 1 | 202 | 202 | 0 | 100 |
| APOB | chr2 | 21260825 | 21260988 | 1 | 164 | 164 | 0 | 100 |
| APOB | chr2 | 21237315 | 21237470 | 1 | 156 | 156 | 0 | 100 |
| APOB | chr2 | 21245698 | 21245919 | 1 | 222 | 222 | 0 | 100 |
| APOB | chr2 | 21263805 | 21263960 | 1 | 156 | 156 | 0 | 100 |
| APOB | chr2 | 21265228 | 21265353 | 1 | 126 | 126 | 0 | 100 |
| APOB | chr2 | 21246392 | 21246569 | 1 | 178 | 178 | 0 | 100 |
| APOB | chr2 | 21241859 | 21241990 | 1 | 132 | 132 | 0 | 100 |
| APOB | chr2 | 21252506 | 21252662 | 1 | 157 | 157 | 0 | 100 |
| APOB | chr2 | 21255221 | 21255458 | 2 | 238 | 238 | 0 | 100 |
| APOB | chr2 | 21227428 | 21227552 | 1 | 125 | 125 | 0 | 100 |
| APOB | chr2 | 21236027 | 21236410 | 2 | 384 | 384 | 0 | 100 |
| APOB | chr2 | 21238237 | 21238422 | 1 | 186 | 186 | 0 | 100 |
| APOB | chr2 | 21239306 | 21239526 | 1 | 221 | 221 | 0 | 100 |
| APOB | chr2 | 21266380 | 21266428 | 1 | 49 | 49 | 0 | 100 |
| APOE | chr19 | 45411012 | 45411214 | 2 | 243 | 243 | 0 | 100 |
| APOE | chr19 | 45409854 | 45409929 | 2 | 116 | 116 | 0 | 100 |
| APOE | chr19 | 45409121 | 45409185 | 2 | 105 | 105 | 0 | 100 |
| APOE | chr19 | 45411785 | 45412512 | 5 | 768 | 768 | 0 | 100 |
| LDLR | chr19 | 11200220 | 11200296 | 1 | 77 | 77 | 0 | 100 |
| LDLR | chr19 | 11227530 | 11227679 | 1 | 150 | 150 | 0 | 100 |
| LDLR | chr19 | 11210894 | 11211026 | 1 | 133 | 133 | 0 | 100 |
| LDLR | chr19 | 11222185 | 11222320 | 1 | 136 | 136 | 0 | 100 |
| LDLR | chr19 | 11230763 | 11230914 | 1 | 152 | 152 | 0 | 100 |
| LDLR | chr19 | 11233845 | 11234025 | 1 | 181 | 181 | 0 | 100 |
| LDLR | chr19 | 11221323 | 11221452 | 1 | 130 | 130 | 0 | 100 |
| LDLR | chr19 | 11226765 | 11226893 | 1 | 129 | 129 | 0 | 100 |
| LDLR | chr19 | 11231041 | 11231203 | 1 | 163 | 163 | 0 | 100 |
| LDLR | chr19 | 11224206 | 11224443 | 2 | 238 | 238 | 0 | 100 |
| LDLR | chr19 | 11218063 | 11218195 | 1 | 133 | 133 | 0 | 100 |
| LDLR | chr19 | 11238679 | 11238766 | 1 | 88 | 88 | 0 | 100 |
| LDLR | chr19 | 11213335 | 11213467 | 1 | 133 | 133 | 0 | 100 |
| LDLR | chr19 | 11215891 | 11216281 | 3 | 391 | 391 | 0 | 100 |
| LDLR | chr19 | 11223949 | 11224130 | 2 | 182 | 182 | 0 | 100 |
| LDLR | chr19 | 11217236 | 11217368 | 1 | 133 | 133 | 0 | 100 |
| LDLR | chr19 | 11240184 | 11240351 | 1 | 168 | 168 | 0 | 100 |

| | | | | | | | | |
|---|---|---|---|---|---|---|---|---|
| LDLR | chr19 | 11241952 | 11241997 | 1 | 46 | 46 | 0 | 100 |
| LIPA | chr10 | 91005428 | 91005555 | 1 | 168 | 168 | 0 | 100 |
| LIPA | chr10 | 90974580 | 90974823 | 2 | 284 | 284 | 0 | 100 |
| LIPA | chr10 | 90984844 | 90984990 | 2 | 187 | 187 | 0 | 100 |
| LIPA | chr10 | 91007290 | 91007410 | 1 | 161 | 161 | 0 | 100 |
| LIPA | chr10 | 90982263 | 90982344 | 1 | 122 | 122 | 0 | 100 |
| LIPA | chr10 | 90975690 | 90975771 | 1 | 122 | 122 | 0 | 100 |
| LIPA | chr10 | 90983436 | 90983592 | 2 | 197 | 197 | 0 | 100 |
| LIPA | chr10 | 90986647 | 90986766 | 2 | 160 | 160 | 0 | 100 |
| LIPA | chr10 | 90987952 | 90988160 | 2 | 249 | 249 | 0 | 100 |
| LDLRAP1 | chr1 | 25889130 | 25889212 | 2 | 123 | 123 | 0 | 100 |
| LDLRAP1 | chr1 | 25870185 | 25870282 | 1 | 138 | 138 | 0 | 100 |
| LDLRAP1 | chr1 | 25881346 | 25881468 | 1 | 163 | 163 | 0 | 100 |
| LDLRAP1 | chr1 | 25891659 | 25891703 | 1 | 85 | 85 | 0 | 100 |
| LDLRAP1 | chr1 | 25893334 | 25893488 | 2 | 195 | 195 | 0 | 100 |
| LDLRAP1 | chr1 | 25890147 | 25890287 | 1 | 181 | 181 | 0 | 100 |
| LDLRAP1 | chr1 | 25880408 | 25880560 | 2 | 193 | 193 | 0 | 100 |
| LDLRAP1 | chr1 | 25883639 | 25883763 | 2 | 165 | 165 | 0 | 100 |
| LDLRAP1 | chr1 | 25889556 | 25889649 | 2 | 134 | 134 | 0 | 100 |
| PCSK9 | chr1 | 55523704 | 55523887 | 1 | 184 | 184 | 0 | 100 |
| PCSK9 | chr1 | 55525154 | 55525341 | 1 | 188 | 188 | 0 | 100 |
| PCSK9 | chr1 | 55517946 | 55518089 | 1 | 144 | 144 | 0 | 100 |
| PCSK9 | chr1 | 55518318 | 55518469 | 1 | 152 | 152 | 0 | 100 |
| PCSK9 | chr1 | 55509511 | 55509712 | 1 | 202 | 202 | 0 | 100 |
| PCSK9 | chr1 | 55521661 | 55521867 | 2 | 207 | 207 | 0 | 100 |
| PCSK9 | chr1 | 55522999 | 55523192 | 1 | 194 | 194 | 0 | 100 |
| PCSK9 | chr1 | 55527043 | 55527234 | 1 | 192 | 192 | 0 | 100 |
| PCSK9 | chr1 | 55505506 | 55505722 | 2 | 217 | 217 | 0 | 100 |
| PCSK9 | chr1 | 55512191 | 55512324 | 1 | 134 | 134 | 0 | 100 |
| PCSK9 | chr1 | 55529037 | 55529262 | 2 | 226 | 226 | 0 | 100 |
| PCSK9 | chr1 | 55524167 | 55524325 | 2 | 159 | 159 | 0 | 100 |

**Table S2: Overall coverage of the main familial hypercholesterolemia associated genes.**

| Gene | Chr | Num Exons | Num Amplicons | Total Bases | Covered Bases | Missed Bases | Overall Coverage (%) |
|---|---|---|---|---|---|---|---|
| *APOB* | chr2 | 29 | 85 | 13982 | 13982 | 0 | 100 |
| *LDLR* | chr19 | 18 | 21 | 2763 | 2763 | 0 | 100 |
| *PCSK9* | chr1 | 12 | 16 | 2199 | 2199 | 0 | 100 |
| *APOE* | chr19 | 4 | 11 | 1232 | 1232 | 0 | 100 |
| *LDLRAP1* | chr1 | 9 | 14 | 1377 | 1377 | 0 | 100 |
| *LIPA* | chr10 | 9 | 14 | 1650 | 1650 | 0 | 100 |

**Table S3: Clinical characteristics of the cohort.**

| Patients | Smoker | DM | HBP | DL | BMI | FH | Highest LDLc |
|---|---|---|---|---|---|---|---|
| Woman 1 | 1 | 0 | 0 | 0 | 30,42 | 0 | 65 |
| Woman 2 | 1 | 1 | 0 | 1 | 33,06 | 1 | 149 |
| Woman 3 | 1 | 0 | 1 | 1 | 34,95 | 0 | 150 |
| Woman 4 | 1 | 0 | 1 | 0 | 23,44 | 0 | 141 |
| Woman 5 | 1 | 0 | 0 | 0 | 24,44 | 1 | 115 |
| Woman 6 | 1 | 0 | 0 | 0 | 28,91 | 0 | 67 |
| Woman 7 | 1 | 0 | 0 | 0 | 31,22 | 1 | 97 |
| Woman 8 | 1 | 0 | 0 | 1 | 21,78 | 0 | 106 |
| Man 1 | 1 | 0 | 0 | 0 | 29,05 | 0 | 148 |
| Man 2 | 1 | 0 | 1 | 0 | 34,06 | 1 | 103 |
| Man 3 | 0 | 0 | 1 | 0 | 27,76 | 0 | 109 |
| Man 4 | 1 | 0 | 1 | 0 | 32,95 | 1 | 188 |
| Man 5 | 0 | 0 | 0 | 0 | 26,3 | 1 | 98 |

| | | | | | | | |
|---|---|---|---|---|---|---|---|
| Man 6 | 1 | 0 | 0 | 0 | 33,53 | 0 | 136 |
| Man 7 | 1 | 0 | 1 | 1 | 32,79 | 0 | 115 |
| Man 8 | 1 | 1 | 1 | 1 | 33,24 | 1 | 217 |
| Man 9 | 0 | 0 | 0 | 1 | 27,04 | 0 | 179 |
| Man 10 | 1 | 1 | 1 | 0 | 27,76 | 0 | 79 |
| Man 11 | 1 | 0 | 0 | 0 | - | 0 | 75 |
| Man 12 | 1 | 0 | 0 | 1 | 25,95 | 0 | 201 |
| Man 13 | 1 | 0 | 0 | 1 | 23,44 | 0 | 207 |
| Man 14 | 1 | 1 | 1 | 1 | 30,8 | 0 | 118 |
| Man 15 | 1 | 0 | 0 | 0 | 33,63 | 0 | 117 |
| Man 16 | 0 | 0 | 0 | 0 | 24,22 | 1 | 113 |
| Man 17 | 1 | 0 | 0 | 1 | 34,72 | 1 | 137 |
| Man 18 | 0 | 0 | 0 | 0 | 28,38 | 1 | 82 |
| Man 19 | 1 | 1 | 0 | 0 | 29,07 | 0 | 137 |
| Man 20 | 1 | 0 | 0 | 0 | 24,26 | 0 | 173 |
| Man 21 | 1 | 0 | 0 | 1 | 27,38 | 0 | 161 |
| Man 22 | 1 | 0 | 1 | 0 | 34,6 | 1 | 118 |
| Man 23 | 1 | 0 | 0 | 1 | 26,12 | 0 | 177 |
| Man 24 | 1 | 0 | 0 | 0 | 26,69 | 1 | 142 |
| Man 25 | 0 | 0 | 0 | 0 | 24,22 | 0 | 80 |
| Man 26 | 1 | 0 | 1 | 0 | 29,41 | 0 | 129 |
| Man 27 | 1 | 0 | 1 | 0 | 27,68 | 0 | 190 |
| Man 28 | 1 | 0 | 1 | 1 | 31,02 | 0 | 174 |
| Man 29 | 1 | 0 | 0 | 0 | 31,79 | 0 | 79 |
| Man 30 | 1 | 0 | 1 | 1 | 29,06 | 0 | 178 |
| Man 31 | 1 | 0 | 0 | 0 | 33,22 | 0 | 89 |
| Man 32 | 0 | 0 | 0 | 0 | 35,51 | 0 | 110 |
| Man 33 | 1 | 0 | 0 | 0 | 22,27 | 0 | 100 |
| Man 34 | 1 | 0 | 0 | 1 | 26,02 | 1 | 138 |
| Man 35 | 1 | 0 | 0 | 0 | 31,14 | 0 | 109 |
| Man 36 | 1 | 0 | 0 | 0 | 20,96 | 0 | 138 |
| Man 37 | 1 | 0 | 0 | 0 | 32,45 | 0 | 118 |
| Man 38 | 1 | 0 | 1 | 1 | 33,02 | 1 | 165 |
| Man 39 | 1 | 1 | 0 | 0 | 30,67 | 0 | 128 |
| Man 40 | 1 | 0 | 0 | 0 | 25,47 | 1 | 164 |
| Man 41 | 1 | 0 | 0 | 1 | 27,75 | 0 | 197 |
| Man 42 | 1 | 0 | 0 | 1 | 27,13 | 0 | 143 |
| Man 43 | 1 | 0 | 1 | 0 | 32,28 | 0 | 129 |
| Man 44 | 1 | 0 | 0 | 1 | 32,51 | 1 | 184 |
| Man 45 | 0 | 0 | 0 | 0 | 29,41 | 0 | 94 |
| Man 46 | 1 | 0 | 0 | 1 | 26,12 | 0 | 152 |
| Man 47 | 1 | 0 | 0 | 1 | 25,95 | 0 | 205 |
| Man 48 | 1 | 0 | 0 | 1 | 26,23 | 0 | 149 |

High blood pressure = HBP; DM = diabetes mellitus; DL = dyslipidemia; BMI = body mass index; FH = familial history of premature CVD; LDLc = LCL cholesterol; highest LDLc = highest available determination of untreated LDLc levels or corrected by lipid-lowering meditation and its dose.

**Table S4: DLCN Score distribution.**

| Patients | Clinical DLCN Score | Family history | Personal clinical history | Physical examination | LDLc punctuation | DNA analysis |
|---|---|---|---|---|---|---|
| Woman 1 | 2 | 0 | 2 | 0 | 0 | negative |
| Woman 2 | 3 | 1 | 2 | 0 | 0 | negative |
| Woman 3 | 2 | 0 | 2 | 0 | 0 | negative |
| Woman 4 | 2 | 0 | 2 | 0 | 0 | negative |
| Woman 5 | 3 | 1 | 2 | 0 | 0 | negative |
| Woman 6 | 2 | 0 | 2 | 0 | 0 | negative |

| | | | | | | |
|---|---|---|---|---|---|---|
| Woman 7 | 3 | 1 | 2 | 0 | 0 | negative |
| Woman 8 | 2 | 0 | 2 | 0 | 0 | negative |
| Man 1 | 2 | 0 | 2 | 0 | 0 | negative |
| Man 2 | 3 | 1 | 2 | 0 | 0 | negative |
| Man 3 | 2 | 0 | 2 | 0 | 0 | negative |
| Man 4 | 4 | 1 | 2 | 0 | 1 | negative |
| Man 5 | 3 | 1 | 2 | 0 | 0 | APOB p.Phe3150Leu |
| Man 6 | 2 | 0 | 2 | 0 | 0 | negative |
| Man 7 | 2 | 0 | 2 | 0 | 0 | negative |
| Man 8 | 6 | 1 | 2 | 0 | 3 | LDLR p.Asp221Gly |
| Man 9 | 3 | 0 | 2 | 0 | 1 | negative |
| Man 10 | 4 | 0 | 2 | 0 | 0 | negative |
| Man 11 | 2 | 0 | 2 | 0 | 0 | negative |
| Man 12 | 5 | 0 | 2 | 0 | 3 | negative |
| Man 13 | 5 | 0 | 2 | 0 | 3 | negative |
| Man 14 | 2 | 0 | 2 | 0 | 0 | negative |
| Man 15 | 2 | 0 | 2 | 0 | 0 | negative |
| Man 16 | 3 | 1 | 2 | 0 | 0 | negative |
| Man 17 | 3 | 1 | 2 | 0 | 0 | negative |
| Man 18 | 3 | 1 | 2 | 0 | 0 | negative |
| Man 19 | 2 | 0 | 2 | 0 | 0 | negative |
| Man 20 | 3 | 0 | 2 | 0 | 1 | negative |
| Man 21 | 3 | 0 | 2 | 0 | 1 | negative |
| Man 22 | 4 | 1 | 2 | 0 | 0 | negative |
| Man 23 | 3 | 0 | 2 | 0 | 1 | negative |
| Man 24 | 3 | 1 | 2 | 0 | 0 | negative |
| Man 25 | 2 | 0 | 2 | 0 | 0 | negative |
| Man 26 | 2 | 0 | 2 | 0 | 0 | negative |
| Man 27 | 5 | 0 | 2 | 0 | 3 | negative |
| Man 28 | 3 | 0 | 2 | 0 | 1 | negative |
| Man 29 | 2 | 0 | 2 | 0 | 0 | negative |
| Man 30 | 3 | 0 | 2 | 0 | 1 | negative |
| Man 31 | 3 | 0 | 2 | 0 | 0 | negative |
| Man 32 | 2 | 0 | 2 | 0 | 0 | negative |
| Man 33 | 4 | 0 | 2 | 0 | 0 | negative |
| Man 34 | 3 | 1 | 2 | 0 | 0 | negative |
| Man 35 | 2 | 0 | 2 | 0 | 0 | negative |
| Man 36 | 2 | 0 | 2 | 0 | 0 | negative |
| Man 37 | 2 | 0 | 2 | 0 | 0 | negative |
| Man 38 | 4 | 1 | 2 | 0 | 1 | negative |
| Man 39 | 2 | 0 | 2 | 0 | 0 | negative |
| Man 40 | 4 | 1 | 2 | 0 | 1 | negative |
| Man 41 | 5 | 0 | 2 | 0 | 3 | negative |
| Man 42 | 2 | 0 | 2 | 0 | 0 | negative |
| Man 43 | 2 | 0 | 2 | 0 | 0 | negative |
| Man 44 | 4 | 1 | 2 | 0 | 1 | negative |
| Man 45 | 2 | 0 | 2 | 0 | 0 | negative |
| Man 46 | 2 | 0 | 2 | 0 | 0 | negative |
| Man 47 | 5 | 0 | 2 | 0 | 3 | negative |
| Man 48 | 2 | 0 | 2 | 0 | 0 | negative |

DLCN= Dutch Lipid Clinic Network; LDLc= LCL cholesterol; LDLc punctuation= 155-189 mg/dL, 1 point; 190-249 mg/dL, 3 points; 250-329 mg/dL, 5 points; >329, 8 points.
